# Novel Computational Approaches and Applications in Cancer Research

**DOI:** 10.1155/2017/9509280

**Published:** 2017-03-21

**Authors:** Min Zhang, Lin Hua, Weiwei Zhai, Yichuan Zhao

**Affiliations:** ^1^Department of Statistics, Purdue University, West Lafayette, IN, USA; ^2^School of Biomedical Engineering, Capital Medical University, Beijing 100069, China; ^3^Genome Institute of Singapore, Singapore; ^4^Department of Mathematics and Statistics, Georgia State University, Atlanta, GA, USA

Cancer remains one of the leading causes of global morbidity and mortality. As an enormous health burden worldwide, cancer touches every geographic region and is growing at an alarming pace. It is projected that 21.7 million new cases and 13.0 million deaths will occur in 2030 alone. To tackle this vicious disease effectively, a concerted effort by both research and healthcare communities is required to yield significant advances in cancer research and therapy. With the recent developments of high-throughput biotechnologies for genomes, proteomes, and transcriptomes, it is essential to develop innovative computational methods for comprehensive data analysis to improve our understanding of cancer initiation, progression, and metastasis. Therefore, novel computational and statistical methods are needed to analyze each type of omics data, to integrate multiple types of data across platforms, and to discover potential cancer-related biomarkers that can shed light on early detection, monitor disease progression, and eventually facilitate the development of personalized therapy of cancer.

The articles contained in the present issue include basic scientific studies focused on novel computational approaches and tools to analyze high-throughput multiplatform cancer data. In addition, image-based biomarkers were also discussed for early detection of the disease.

Diagnosis of tumor and definition of tumor borders intraoperatively are primarily based on the visualization modalities. However, intraoperative fast histopathology is often not sufficient. The contribution by A. Kamen et al. in “Automatic Tissue Differentiation Based on Confocal Endomicroscopic Images for Intraoperative Guidance in Neurosurgery” proposes an automated endomicroscopic tissue differentiation algorithm based on the machine learning theory. This algorithm offers a useful component to an intraoperative pathology system for guiding the resection procedure based on cellular level information.

Breast cancer is one of the most commonly diagnosed cancers in women all over the world. Osteopontin (OPN) is overexpressed in breast cancers, while its clinical and prognostic significance remain unclear. The contribution by C. Hao et al. in “Prognostic Value of Osteopontin Splice Variant-c Expression in Breast Cancers: A Meta-Analysis” proposes assessing the prognostic value of OPN, especially its splice variants, in breast cancers from eligible studies concerning the OPN and OPN-c expression. It concludes that the high level of OPN-c is suggested to be more reliably associated with poor survival in breast cancer patients.

Apolipoprotein E* (ApoE)*  *ε*4 allele has been proved to be a risk gene of late-onset Alzheimer's disease. It is very important to look for sensitive and reliable biomarkers in earliest stages. In the paper by Y. Liang “Frequency Specific Effects of* ApoE ε*4 Allele on Resting-State Networks in Nondemented Elders,” the authors applied resting-state functional magnetic resonance imaging to examine the* ApoE ε*4 allele effects on functional connectivity of the default mode network and the salience network (SN). They conclude a frequency dependent effect of resting-state signals when investigating resting-state networks functional connectivity.

With the accumulation of large scale omic data, finding genomic features (variables) associated with clinical outcomes is an important topic for precision medicine. Variable selection provides a powerful tool for this practical need. In the manuscript entitled “High Dimensional Variable Selection with Error Control,” S. Kim and S. Halabi present a sequential method based on false discovery rate (FDR) and iterative sure independence screening (ISIS). On the basis of both simulation study and real data analyses, the authors demonstrate the utility and statistical properties of the new method.

Finding correlation between epigenetic changes and certain phenotypes of interest is an important and hotly debated topic. In the article entitled “An Efficient Approach to Screening Epigenome-Wide Data,” M. A. Ray et al. propose an improved and more efficient screening method to look for (filter) informative DNA methylation sites. By incorporating surrogate variable analysis (SVA) and identifying unknown latent variables, the proposed method shows superior performance in identifying epigenetic changes associated with maternal smoking. The developed method has been implemented into an efficient and user-friendly R package.

We hope that the methods proposed in this special issue could help discover potential cancer-related biomarkers or therapy targets and facilitate the biological experiments and biological technology development for cancer-related research.

